# A Biomimetic Amelogenin–Fibronectin Fusion Protein with Dual Cell-Adhesive and Osteoinductive Functions for Alveolar Bone Defect Repair

**DOI:** 10.3390/bioengineering13060646

**Published:** 2026-05-30

**Authors:** Mengsong Zheng, Xinyi Jiang, Minghua Lei, Bin Liang, Xiaoshuang Ye, Lining Xie, Peirong Zhang, Yili Li, An Hong, Zhijian Su, Xiaojia Chen

**Affiliations:** 1 Department of Cell Biology, College of Life Science and Technology, Jinan University, Guangzhou 510632, China; mengsongzheng@163.com (M.Z.); xinyijiang01@163.com (X.J.); minghua_lei01@163.com (M.L.); 13660624543@163.com (B.L.); yxs15224726021@163.com (X.Y.); asdfwqeljk@163.com (L.X.); 13519794002@163.com (P.Z.); 18687611911@163.com (Y.L.); 2 National Engineering Research Center of Genetic Medicine, Guangzhou 510632, China; 3 Guangdong Province Key Laboratory of Bioengineering Medicine, Guangzhou 510632, China; 4 Guangdong Provincial Biotechnology Drug & Engineering Technology Research Center, Guangzhou 510632, China; 5 School of Pharmaceutical Sciences, Wenzhou Medical University, Wenzhou 325000, China; 6 National Engineering Research Center for Cell Growth Factor Drugs and Protein Biologics, Wenzhou 325000, China; 7 National Key Laboratory of Macromolecular Drug Development and Manufacturing, Wenzhou 325000, China

**Keywords:** alveolar bone regeneration, amelogenin, RGD domain, cell adhesion

## Abstract

Periodontitis-induced destruction of periodontal tissues and tooth loss remain major clinical challenges. Although periodontal regenerative therapies aim to reconstruct damaged structures, particularly the repair of alveolar bone defects, current biomaterials have limited capacity to simultaneously promote cell adhesion and osteogenic differentiation. Amelogenin (Am) plays a key role in mineralized tissue development through its highly conserved N-terminal and C-terminal regions, but studies have shown that Am has certain limitations in promoting cell adhesion. In contrast, the arginine-glycine-aspartic acid (RGD) domain of fibronectin (FN) effectively mediates cell–matrix adhesion. Based on these properties, we developed a novel recombinant fusion protein (rtAmR) by combining the conserved regions of Am with the RGD domain of FN. In vitro, rtAmR significantly promoted the adhesion and spreading of human stem cells from the apical papilla (hSCAPs) compared with the control group. Quantitative analysis showed that the number of adherent cells and the cell spreading area in the rtAmR group were 1.9-fold and 2.1-fold higher than those in the rhAm group, respectively. In osteogenic differentiation assays, rtAmR exhibited activity comparable to that of rhAm and even outperformed rhAm in terms of alkaline phosphatase (ALP) activity, collagen type I (COL I) expression, and calcium nodule formation. In a Sprague–Dawley (SD) rat alveolar bone defect model, rtAmR treatment significantly promoted bone regeneration, achieving superior bone volume/total volume (BV/TV) values compared to the rhAm and rhFN groups. Immunohistochemistry revealed that rtAmR did not obviously increase neutrophils, mast cells, or M2 macrophages versus control, confirming its biosafety and suggesting M2-independent osteogenesis. These findings suggest that rtAmR is a promising bifunctional bioactive protein for periodontal bone regeneration.

## 1. Introduction

Periodontitis is a chronic inflammatory disease induced by pathogenic bacterial infection, clinically characterized by progressive destruction of periodontal supporting tissues and continuous alveolar bone loss [1,2]. In the 2021 Global Burden of Disease study, severe periodontitis affected over 1.06 billion people globally (age-standardized prevalence 12.50%), projected to exceed 1.56 billion by 2050 [3]. The ultimate goal of periodontal therapy is the regeneration of periodontal tissues, particularly the restoration of alveolar bone defects [4]. However, conventional therapeutic approaches mainly focus on infection control, such as scaling and root planing, and do not adequately reconstruct severely damaged tissues [5,6,7]. Although guided tissue regeneration (GTR), bone graft substitutes, and growth factor–based therapies have been introduced into clinical practice, their broader application remains limited by narrow indications, insufficient bioactivity, or high cost [8,9,10,11]. Therefore, developing bioactive materials that can actively regulate cellular behavior and promote efficient bone regeneration is crucial for periodontal tissue engineering.

Periodontal bone regeneration is a multistep and highly coordinated biological process involving early cell adhesion, osteogenic differentiation, and late-stage extracellular matrix mineralization, which collectively depend on cell recruitment, adhesion, proliferation, and differentiation [12,13]. In this context, the extracellular matrix (ECM) serves not only as a structural scaffold but also as a dynamic signaling platform that modulates cell behavior through specific biochemical and biomechanical cues [14]. Early cell–ECM interactions can induce actin cytoskeleton reorganization and intracellular tension generation, thereby activating osteogenesis-related transcription factors and initiating mineralized tissue formation [15]. Consequently, biomimetic ECM-inspired designs have emerged as an effective strategy to enhance the functional performance of regenerative biomaterials.

Amelogenin (Am) serves as the major protein component in the enamel matrix and exerts a vital function in the development and regeneration of periodontal tissues [16]. Emdogain^®^, an enamel matrix derivative that has been clinically applied, has been proven to facilitate the regeneration of cementum and alveolar bone, where enamel matrix proteins act as its main bioactive components [17]. In terms of structure, Am is composed of a tyrosine-enriched N-terminal domain, a central hydrophobic region, and a hydrophilic C-terminal domain [18]. Numerous studies have indicated that the bioactivity of Am is primarily derived from its highly conserved N-terminal and C-terminal domains, which participate in regulating the nucleation of hydroxyapatite and the orientation of crystals [19]. Notably, the naturally occurring splice variant, leucine-rich Am peptide (LRAP), which does not contain the central hydrophobic region, still maintains robust bioactivity and can effectively induce the osteogenic differentiation of mesenchymal stem cells [20,21]. These research findings lay a structural foundation for the rational design and modification of bioactive molecules derived from Am.

Despite its promising osteoinductive potential, Am exhibits limited performance in promoting early cell adhesion, which may restrict its regenerative efficiency [22,23]. Fibronectin (FN) is a key ECM glycoprotein responsible for mediating cell adhesion, and its arginine-glycine-aspartic acid (RGD) domain serves as a principal binding site for integrins such as α5β1 [24,25,26]. Through integrin-mediated binding, the RGD domain establishes robust cell–matrix interactions and triggers intracellular signaling cascades essential for cell survival, migration, and lineage commitment [27].

**Figure 1:**
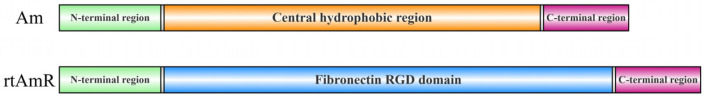
Schematic diagram of the primary structures of Am and rtAmR.

Based on these considerations, we rationally designed a novel recombinant fusion protein (rtAmR) using a biomimetic and function-oriented engineering strategy. This fusion protein preserves the highly bioactive N- and C-terminal conserved domains of Am, while the central hydrophobic region is replaced with the RGD domain derived from FN, thereby integrating osteoinductive and cell-adhesive functionalities into a single molecule. The comparative primary structures of the native Am and the engineered rtAmR are schematically presented in Figure 1. rtAmR was prepared using an *E. coli* expression system. We then evaluated its effects on cell adhesion and osteogenic differentiation in vitro using human stem cells from the apical papilla (hSCAPs), and further validated its reparative efficacy in vivo using a rat alveolar bone defect model. Immunohistochemical analysis of neutrophils, mast cells, and M2 macrophages was performed to assess the biosafety of rtAmR and to explore whether its osteogenic effect depends on M2-mediated immunomodulation. Collectively, this study provides a novel biological strategy for engineering bioactive proteins for the treatment of periodontal bone defects.

## 2. Materials and Methods

### 2.1. Materials

The rhFN with cell adhesion properties was acquired from the Biopharmaceutical Research & Development Center at Jinan University (Guangzhou, China) [28]. rhAm was synthesized via a recombinant expression system within the Institute of Bioengineering, Jinan University (Guangzhou, China). The hSCAPs were sourced from the School of Stomatology at Jinan University (Guangzhou, China). Male Sprague–Dawley (SD) rats were procured from Beijing Vital River Laboratory Animal Technology Co., Ltd. (Beijing, China) and maintained in a specific pathogen-free (SPF) environment at the Laboratory Animal Center of Jinan University (Guangzhou, China).

### 2.2. Construction and Transformation of the rtAmR Expression Vector

The rtAmR-encoding cDNA sequence was codon-optimized for E. coli expression and was produced by Genewiz Biotechnology Co., Ltd. (Suzhou, China). Afterwards, this optimized fragment was cloned into the pET-28a expression backbone utilizing BamHI and XhoI restriction endonuclease sites, producing the recombinant pET(rtAmR) plasmid. The resulting construct was then transferred into DH5α competent cells (Sangon Biotech, Shanghai, China) for plasmid amplification. Positive transformants were isolated on agar media containing kanamycin (Macklin, Shanghai, China). and further confirmed by means of colony PCR and DNA sequence analysis using the primers listed in Table 1. Upon sequence authentication, the purified plasmid was introduced into BL21(DE3) (Sangon Biotech, Shanghai, China) for the purpose of protein expression. The obtained recombinant strains were preserved for future protein induction studies.

**Table 1:** The primer sequences for colony PCR.

Primer Name	Primer Sequences (5′–3′)
pET(rtAmR)-F	CCATGAAAAGCAGCCATCACCAT
pET(rtAmR)-R	GTTAATCCACTTCTTCGCGTTTGGT

### 2.3. Expression, Purification, and Characterization of rtAmR

Glycerol stocks of the engineered strain were seeded at a 1:100 ratio into kanamycin-supplemented LB broth and incubated at 37 °C with shaking (220 rpm) for 12 h. The culture was then inoculated at a 5% ratio into fresh LB medium containing kanamycin and grown until the OD_600_ reached 0.8–1.2. To trigger protein production, 1 mM isopropyl β-D-1-thiogalactopyranoside (IPTG, GBCBIO Technologies Inc., Guangzhou, China) was added, followed by incubation at 37 °C for 4 h. The bacteria were then collected via centrifugation, reconstituted in chilled binding buffer, and disrupted with a high-pressure microfluidizer at 4 °C until the mixture cleared. After centrifuging the lysate to isolate the supernatant, the cellular debris pellet was saved in PBS for subsequent evaluation. The target rtAmR was isolated from the soluble phase utilizing a HisTrap HP chromatography column (Cytiva, Marlborough, MA, USA). Both the flow-through fractions and the eluates from an imidazole gradient were gathered during this procedure. Stepwise samples underwent SDS-PAGE followed by Coomassie Brilliant Blue staining for initial characterization, while Western blot was utilized to verify rtAmR expression. Finally, the purity of the protein was evaluated via high-performance liquid chromatography (HPLC).

### 2.4. Cell Culture

The hSCAPs were maintained in α-MEM (Gibco, Grand Island, NY, USA) enriched with 10% FBS (PAN-Biotech, Aidenbach, Germany) within a humidified incubator at 37 °C and 5% CO_2_. Once the cells attained roughly 80% confluence, they were washed with PBS and dissociated using 0.25% trypsin-EDTA (Gibco, Grand Island, NY, USA) for 40 s at 37 °C. The enzymatic reaction was terminated via the addition of FBS-containing complete medium. Subsequently, the suspension was subjected to centrifugation for 5 min at 1000 rpm, followed by the removal of the supernatant. The resulting cell pellet was reconstituted in fresh complete medium, quantified via a hemocytometer, and plated into new flasks at a suitable density for passaging or downstream assays.

### 2.5. Cytocompatibility Evaluation (CCK-8 Assay)

P3–P5 hSCAPs were seeded into 96-well plates at a density of 3 × 10^3^ cells per well. After 24 h, different concentrations of rtAmR (0, 60, 120, 240, 480, and 960 ng/mL) were added, and the plates were incubated for 24, 48, and 72 h respectively. Then, serum-free α-MEM (Gibco, Grand Island, NY, USA) containing 10% CCK-8 (Selleck, Houston, TX, USA) was added to all wells (including the blank wells) and incubated at 37 °C until the OD value of the control group reached 0.8–1.2. Cell viability was assessed by measuring the OD value at 450 nm. The blank well OD value was used to subtract background interference during the subsequent calculation, and the relative cell activity for each group was calculated as shown in Equation (1). P3–P5 hSCAPs were seeded into 96-well plates at a density of 3 × 10^3^ cells per well. After 24 h, different concentrations of rtAmR (0, 60, 120, 240, 480, and 960 ng/mL) were added, and the plates were incubated for 24, 48, and 72 h respectively. Then, serum-free α-MEM (Gibco, Grand Island, NY, USA) containing 10% CCK-8 was added to all wells (including the blank wells) and incubated at 37 °C until the OD value of the control group reached 0.8–1.2. Cell viability was assessed by measuring the OD value at 450 nm. The blank well OD value was used to subtract background interference during the subsequent calculation, and the relative cell activity for each group was calculated using the following formula:

(1) $$Cell\;viability(\%)=\frac{OD\;of\;treated\;group-OD\;of\;blank}{OD\;of\;control\;group-OD\;of\;blank}\times 100\%$$

### 2.6. Cell Adhesion Assay

For the coating process, 96-well plates were incubated overnight at 4 °C with 100 μL per well of a solution containing 240 ng/mL of either rhFN, rhAm, or rtAmR. PBS-treated wells were utilized as blank controls. Following this step, blocking was performed using 1% bovine serum albumin (BSA, Merck, Darmstadt, Germany) for 1 h at 37 °C. Next, 100 μL of hSCAP suspension (seeded at 5 × 10^3^ cells/well) was introduced and maintained at 37 °C within a humidified 5% CO_2_ incubator for 30 min to facilitate cellular attachment. Unattached cells were eliminated by washing the wells gently three times using pre-warmed PBS. The attached cells were then subjected to fixation using 4% paraformaldehyde for 30 min, followed by staining with a crystal violet solution (Solarbio, Beijing, China) for another 30 min. Residual dye was washed away with PBS rinses until a clear background was achieved.

### 2.7. Cytoskeletal Staining

Tissue culture (TC)-treated glass coverslips (Acmec Biochemical, Shanghai, China) were positioned within 24-well plates and incubated with 500 μL of a coating buffer comprising 240 ng/mL of rhFN, rhAm, or rtAmR. Wells receiving an equivalent volume of PBS were utilized as blank controls. Following an overnight incubation at 4 °C, the plates were blocked using 1% BSA for 1 h at 37 °C. Next, 500 μL of the hSCAP suspension (seeded at 2 × 10^4^ cells/well) was introduced, and the samples were maintained at 37 °C in a 5% CO_2_ environment for 1 h to facilitate cellular adherence. Post-incubation, the culture medium was discarded, and the wells were gently rinsed thrice utilizing pre-warmed PBS. The adhered cells were subsequently subjected to cytoskeletal staining employing a commercially available cytoskeleton staining kit (Biosharp, Hefei, China) in accordance with the manufacturer’s protocol.

### 2.8. Osteogenic Differentiation Induction

hSCAPs (passages 3 to 5) were introduced into 24-well plates at a seeding density of 3 × 10^4^ cells per well and grown in complete medium under standard parameters (37 °C with 5% CO_2_) to near 80% confluence. Following this, the initial medium was substituted with osteogenic induction medium (OriCell, Shanghai, China). The experimental conditions involved supplementing the induction medium with 240 ng/mL rhAm or 240 ng/mL rtAmR, while the control wells were supplied with plain induction medium. Over the course of the induction process, culture media replacements were executed every 3 days. On days 7 and 14, the cultures were terminated, and the cells were gathered for the subsequent assessment of osteogenic differentiation indicators.

### 2.9. Alkaline Phosphatase (ALP) Staining and Activity Assay

After 7 days of osteogenic induction, hSCAPs were evaluated for alkaline phosphatase (ALP) expression via both staining and enzymatic activity tests. Regarding the ALP staining, the cultures underwent fixation with 4% paraformaldehyde (Biosharp, Hefei, China) for 30 min at ambient temperature, followed by staining utilizing a BCIP/NBT ALP kit (Beyotime Biotechnology, Shanghai, China) as per the supplier’s guidelines. The enzymatic activity of ALP was measured utilizing a commercially available assay kit (Beyotime Biotechnology, Shanghai, China). Lysates of the cells were generated, and the corresponding enzyme activity was determined following the recommended procedures. The acquired data were standardized against the total protein content to adjust for differences in cell quantities.

### 2.10. Western Blot Analysis

Following 7 days of osteogenic differentiation, the attached cells were disrupted utilizing RIPA lysis buffer (Beyotime Biotechnology, Shanghai, China) supplemented with protease inhibitors. Following a 15 min centrifugation at 15,000× *g* and 4 °C, the resultant supernatant was harvested, and the overall protein content was quantified employing a BCA protein assay kit (Thermo Scientific, Waltham, MA, USA). Equivalent quantities of protein samples were resolved via SDS-PAGE and electroblotted onto PVDF membranes (Merck, Darmstadt, Germany). After blocking with 5% BSA for 1 h, the membranes were incubated overnight at 4 °C with the anti-COL I primary antibody (Cell Signaling Technology, Danvers, MA, USA), followed by a 1 h incubation at room temperature with HRP-linked secondary antibodies (Sangon Biotech, Shanghai, China). Finally, the targeted protein bands were detected utilizing an enhanced chemiluminescence reagent (Beyotime Biotechnology, Shanghai, China) and captured via a chemiluminescence imaging apparatus.

### 2.11. Quantitative Real-Time PCR (qPCR) Analysis

Following 7 days of osteogenic differentiation, total RNA was extracted from the attached cells using the RNAeasy™ Animal RNA Isolation Kit (Beyotime Biotechnology, Shanghai, China) according to the manufacturer’s instructions. Reverse transcription was then performed using the HiScript IV All-in-One Ultra RT SuperMix for qPCR (Vazyme, Nanjing, China) following the manufacturer’s protocol. Quantitative real-time PCR (qPCR) was carried out with the PerfectStart Universal Green qPCR SuperMix (TransGen Biotech, Beijing, China) as per the manufacturer’s recommendations. The primer sequences for RUNX2, OSX, and the internal control GAPDH are listed in Table 2. The relative expression levels of target genes were calculated using the 2^−ΔΔCt^ method, with normalization to GAPDH.

### 2.12. Alizarin Red S Staining

After 14 days of osteogenic differentiation, the generation of mineralized matrix was evaluated utilizing Alizarin Red S staining. The cultures underwent fixation in 4% paraformaldehyde for 30 min, followed by an incubation with Alizarin Red S dye (OriCell, Suzhou, China) at ambient temperature under mild agitation for 20 min. Following the staining procedure, the culture wells were washed using deionized water until a colorless background was achieved. Photographic records were captured for visual documentation, preceding the quantitative evaluation. To quantify the results, the incorporated stain was extracted using a 10% cetylpyridinium chloride buffer (Macklin, Shanghai, China), and the optical density was recorded at 562 nm to assess the extent of calcium deposition.

### 2.13. In Vivo Functional Evaluation

This study was approved by the Laboratory Animal Management Center of Jinan University (Approval No. 20250915-03). All animal procedures were performed in compliance with the relevant ethical guidelines and regulations. The rats were housed in the animal facilities of the Jinan School of Medicine under controlled light conditions (12 h light/dark cycle) with free access to food and water.

Sixteen 4-week-old male Sprague–Dawley rats were placed under general anesthesia, and a standardized alveolar bone defect was surgically created on the mesial side of the right maxillary first molar. Anesthesia was induced by intraperitoneal injection of 2% sodium pentobarbital (dissolved in PBS) at a dose of 40 mg/kg body weight. The procedure was then performed as follows: a 1 cm incision was made along the mesial gingival sulcus, a full-thickness mucoperiosteal flap was elevated to expose the underlying alveolar bone, and a standardized cylindrical bone defect was prepared using a low-speed round bur under continuous saline irrigation. Subsequently, 20 μL of Matrigel (Corning, NY, USA) was implanted into the bone defect; this volume exactly filled the defect cavity. Matrigel provides a three-dimensional extracellular matrix that mimics the microenvironment, promotes cell infiltration, and prolongs the retention time of injected rtAmR at the defect site. The mucoperiosteal flap was then carefully repositioned and sutured with absorbable sutures. On the first day after surgery, all rats were randomly divided into four groups (n = 4 per group): Control, rhFN, rhAm, and rtAmR. Thereafter, at a fixed time each day, 20 μL of the corresponding reagent (PBS, rhFN, rhAm, or rtAmR) was subcutaneously injected into the defect area using a microsyringe. After four weeks of continuous treatment, the animals were euthanized, and maxillary samples containing the defect sites were harvested for qualitative and quantitative evaluation of bone regeneration using micro-computed tomography (micro-CT).

### 2.14. Histological Analysis

After decalcification utilizing a 10% ethylenediaminetetraacetic acid (EDTA) buffer, the bone samples were washed extensively with tap water, subjected to dehydration via a graded series of ethanol, rendered transparent with xylene, and finally embedded in paraffin wax. Consecutive 5-μm-thick slices were prepared utilizing a microtome, transferred onto glass slides, and baked at 65 °C. For the subsequent histological assessment, the sections were stained employing hematoxylin and eosin (H&E, (Beyotime Biotech nology, Shanghai, China) alongside Masson’s trichrome (Beyotime Biotech nology, Shanghai, China) protocols.

Quantitative analysis of collagen deposition was performed on Masson’s trichrome-stained sections using ImageJ software (version 1.54p, NIH, Bethesda, MD, USA). The Color Deconvolution plugin was utilized to distinguish blue-stained collagen fibers from red-stained muscle fibers and cytoplasmic components. After applying appropriate thresholding, the area of collagen-positive (blue-stained) regions was quantified, and the total tissue area in the same field of view was measured accordingly. The relative collagen content (%) was calculated as the percentage of the collagen-positive area relative to the total tissue area.

### 2.15. Immunohistochemical (IHC) Staining

Paraffin-embedded sections (5 μm) were deparaffinized, rehydrated, and subjected to antigen retrieval in citrate buffer (pH 6.0) using microwave heating. Endogenous peroxidase was blocked with 3% H_2_O_2_, and non-specific binding was blocked with 5% normal goat serum. Sections were incubated overnight at 4 °C with primary antibodies targeting CD117 (mast cell marker, Servicebio, Wuhan, China), MPO (neutrophil marker, Servicebio, Wuhan, China), and CD206 (M2 macrophage marker, Servicebio, Wuhan, China). After washing, HRP-conjugated secondary antibody (Servicebio, Wuhan, China) was applied for 1 h at 37 °C. Staining was visualized with DAB substrate, and sections were counterstained with hematoxylin.

### 2.16. Statistical Analysis

Statistical evaluations were executed utilizing GraphPad Prism 8 software, and the results are presented as the mean ± standard deviation (SD). Every experimental procedure consisted of a minimum of three independent replicates. To evaluate differences across multiple categories, a one-way analysis of variance (ANOVA) was applied, succeeded by Tukey’s honestly significant difference (HSD) post hoc analysis. A *p*-value of less than 0.05 was considered to indicate statistical significance.

## 3. Results

### 3.1. Expression, Purification and Characterization of rtAmR

**Figure 2:**
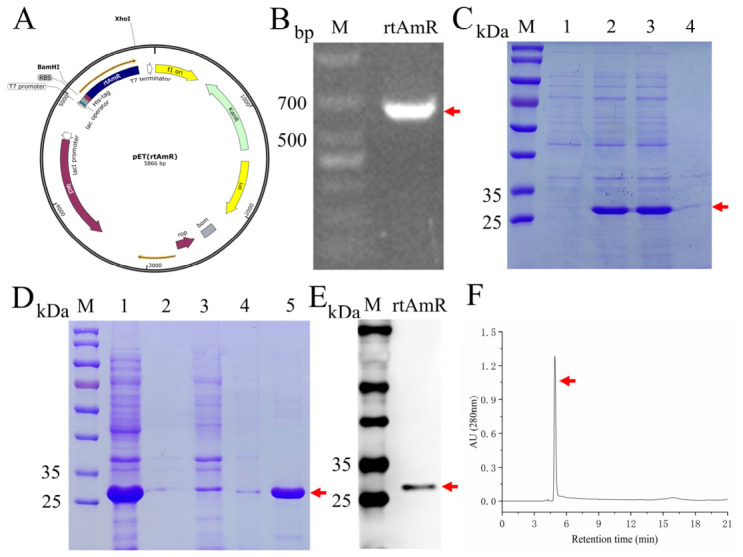
Expression, purification, and characterization of rtAmR. (**A**) Vector map illustrating the pET(rtAmR) construct; (**B**) electrophoretic separation of the PCR-amplified rtAmR on an agarose gel. Lane M: DNA marker (1000 bp); (**C**) SDS-PAGE monitoring of rtAmR induction. Lane M: Molecular weight ladder; Lane 1: Non-induced cell; Lane 2: Induced cell; Lane 3: Supernatant of induced cell; Lane 4: Pellet of induced cell. (**D**) SDS-PAGE demonstrating the rtAmR purification process. Lane M: Molecular weight ladder; Lane 1: Supernatant; Lane 2: Pellet; Lane 3: Flow-through; Lane 4: Wash; Lane 5: Eluted rtAmR. (**E**) Western blot analysis of purified rtAmR utilizing a His-tag specific antibody. Lane M: Molecular weight ladder. (**F**) Purity assessment of rtAmR via HPLC. Red arrows (**B**–**E**): Target bands corresponding to rtAmR; Red arrow (**F**): Characteristic peak of rtAmR.

The recombinant plasmid was constructed and designated as pET(rtAmR), with a 6×His tag sequence introduced at the N terminus to facilitate subsequent protein purification (Figure 2A). Agarose gel electrophoresis confirmed that the size of the target gene fragment matched the theoretically predicted length (Figure 2B). The resulting plasmid was transformed into BL21(DE3), and recombinant protein expression was induced by IPTG. Compared with the uninduced control, a distinct band corresponding to rtAmR was observed in the induced bacterial lysate, and the target protein was predominantly present in the soluble fraction (Figure 2C). rtAmR was purified using Ni-NTA affinity chromatography, and the bound protein was eluted with imidazole-containing buffer (Figure 2D). Western blot analysis using an anti-His tag monoclonal antibody further confirmed the successful expression and purification of rtAmR (Figure 2E). High-performance liquid chromatography (HPLC) analysis revealed that the final purity of the purified rtAmR protein exceeded 92% (Figure 2F).

### 3.2. Biocompatibility of rtAmR

**Figure 3:**
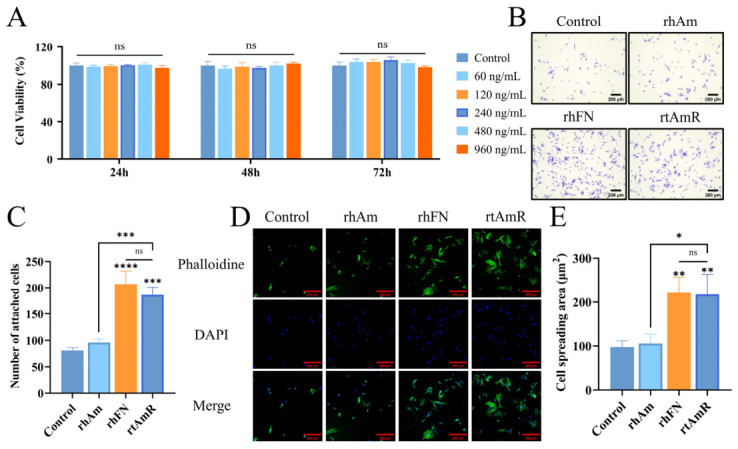
Cytotoxicity and cell-adhesive activity of rtAmR. (**A**) Cytotoxicity of rtAmR on hSCAPs assessed by CCK-8 assay; (**B**) crystal violet staining showing the adhesion of hSCAPs; (**C**) quantification of adherent cell numbers; (**D**) fluorescence staining of the cytoskeleton illustrating the effect of rtAmR on cytoskeletal reorganization in hSCAPs; (**E**) quantitative analysis of cell spreading area. *p* < 0.05 was considered statistically significant. ns represents *p *≥ 0.05, * represents *p* < 0.05, ** represents *p* < 0.01, *** represents *p* < 0.001 and **** represents *p* < 0.0001.

hSCAPs can be harvested from the apical papilla of developing immature permanent teeth. These cells possess multipotent differentiation capabilities and contribute significantly to the formation of dental roots and alveolar bone [29,30,31]. Consequently, hSCAPs were selected as the in vitro cellular model for the current research. The biocompatibility of rtAmR was evaluated via the CCK-8 assay. The findings demonstrated that, within the utilized concentration range, rtAmR exerted no evident cytotoxicity on hSCAPs following a 72 h incubation period (Figure 3A).

### 3.3. Cell Adhesion-Promoting Activity of rtAmR

The adhesion capacity of hSCAPs on rtAmR was examined utilizing crystal violet staining. In comparison with the blank control, both the rhFN- and rtAmR-treated groups displayed a substantial rise in the number of adherent cells, with no marked disparity observed between these two groups. Conversely, the pro-adhesive influence of rtAmR was markedly more potent than that of the rhAm group (Figure 3B). Quantitative evaluation demonstrated that the adherent cell counts in the rhFN and rtAmR groups were 2.5-fold and 2.3-fold higher than those of the control, respectively; notably, rtAmR treatment resulted in a 1.9-fold elevation compared to the rhAm group (Figure 3C). Furthermore, cytoskeletal visualization confirmed that cells exposed to rhFN or rtAmR exhibited a significantly expanded spreading area relative to the control group (Figure 3D). Additionally, the spreading area of cells stimulated by rtAmR was 2.1 times that of the rhAm group (Figure 3E). Taken together, these findings suggest that rtAmR enhances cellular adhesion with an efficiency similar to rhFN and significantly exceeding that of rhAm.

### 3.4. Osteogenic Differentiation Potential of rtAmR

**Figure 4:**
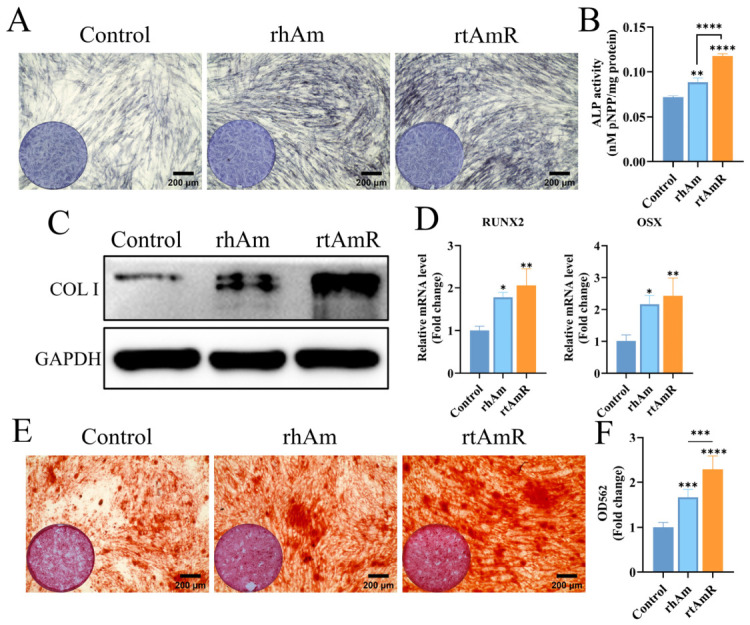
Analysis of the osteogenic differentiation-promoting bioactivity of rtAmR. (**A**) ALP staining to validate the osteogenic differentiation-promoting activity of rtAmR; (**B**) ALP activity assay to confirm the osteogenic differentiation-promoting activity of rtAmR; (**C**) expression levels of COL I following osteogenic differentiation induction; (**D**) expression levels of RUNX2 and OSX mRNA following osteogenic differentiation induction; (**E**) Alizarin Red S staining to detect the formation of calcium nodules; (**F**) quantitative analysis measured at OD_562_. *p* < 0.05 was considered statistically significant. * represents *p* < 0.05, ** represents *p* < 0.01, *** represents *p* < 0.001 and **** represents *p* < 0.0001.

ALP and mineralized nodules are widely used as phenotypic markers for early and late osteoblast differentiation, respectively [32,33]. To investigate the effect of rtAmR on the osteogenic differentiation of hSCAPs in vitro, we performed ALP staining, ALP activity assays and Alizarin Red S staining. ALP staining showed that both rhAm and rtAmR treatments markedly increased staining intensity relative to the control group (Figure 4A). Quantitative ALP activity assays corroborated these observations, revealing significantly elevated ALP activity in both treatment groups, with the rtAmR group exhibiting higher activity than the rhAm group (Figure 4B). Western blot analysis demonstrated that rhAm and rtAmR significantly upregulated the expression of osteogenic marker COL I, an effect that was more pronounced in the rtAmR group (Figure 4C). qPCR analysis revealed that rtAmR treatment significantly upregulated the mRNA levels of the key osteogenic transcription factors RUNX2 and OSX by 2.1-fold and 2.4-fold, respectively, compared with the control group (Figure 4D). Alizarin Red S staining and subsequent quantification indicated that both treatments enhanced calcium nodule formation, with the rtAmR group showing significantly greater mineral deposition than the rhAm group (Figure 4E,F). Collectively, these findings demonstrate that the engineered fusion protein rtAmR retains the capacity to promote osteogenic differentiation in hSCAPs.

### 3.5. The Promotive Effect of rtAmR on Periodontal Bone Regeneration

**Figure 5:**
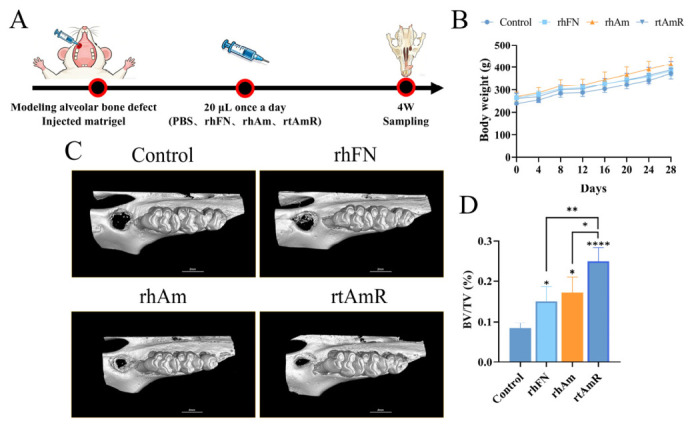
Construction of the SD rat periodontal bone defect model and Micro-CT assessment of periodontal repair. (**A**) Schematic diagram depicting the animal experimental timeline; (**B**) variations in body weight of the rats across all groups during the study period; (**C**) typical three-dimensional Micro-CT reconstructions of the periodontal defect sites; (**D**) quantitative evaluation of bone regeneration parameters via Micro-CT analysis. *p* < 0.05 was considered statistically significant. * represents *p* < 0.05, ** represents *p* < 0.01, and **** represents *p* < 0.0001.

To assess the in vivo osteogenic capacity of rtAmR, a critical-sized periodontal bone defect was established in the maxillae of SD rats [34]. The schematic representation of the animal experimental protocol is depicted in Figure 5A. Throughout the study, body weight monitoring revealed no substantial weight loss across any of the groups (Figure 5B). Micro-CT imaging illustrated that new bone formation initiated from the defect peripheries and progressed toward the center in all experimental groups. In comparison to the control, bone regeneration was markedly improved in the rhFN, rhAm, and rtAmR groups, with the rtAmR group displaying the most prominent osseous healing within the defect area (Figure 5C). Quantitative evaluation of the bone volume fraction (BV/TV) showed a lower baseline in the control group (8.4% ± 1.3%), whereas significant elevations were noted in the rhFN (15.1% ± 3.5%), rhAm (17.1% ± 3.9%), and rtAmR (24.9% ± 3.4%) groups. Notably, the rtAmR group achieved the highest BV/TV value among all treatment cohorts (Figure 5D).

**Figure 6:**
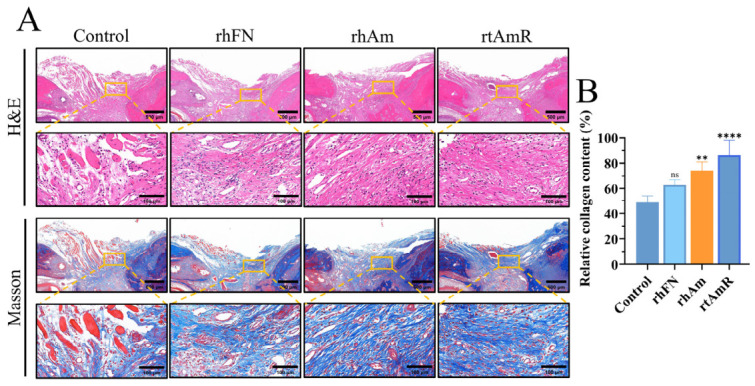
Histological analysis of alveolar bone defect repair in rats. (**A**) H&E and Masson’s trichrome-stained histological sections of decalcified rat maxillae. Regions marked by yellow rectangles are displayed at higher magnification in the panels to the right. (**B**) Quantitative analysis of collagen proportion in newly formed tissue. *p* < 0.05 was considered statistically significant. ns represents *p* ≥ 0.05, ** represents *p* < 0.01, and **** represents *p* < 0.0001.

H&E staining revealed newly formed tissue within the defect area in all groups, with the rhAm and rtAmR groups exhibiting a more compact tissue architecture (Figure 6A). Collagen fiber regeneration was assessed by Masson’s trichrome staining. Compared with the control group, the rhFN, rhAm, and rtAmR groups all displayed more distinct blue collagen networks, indicating the presence of mature collagen fibers within the newly formed bone. Moreover, collagen deposition was notably higher in the rtAmR group than in the rhFN and rhAm groups (Figure 6A). Quantitative results demonstrated that the relative collagen content in the rhFN, rhAm, and rtAmR groups was 1.29-fold, 1.53-fold, and 1.77-fold that of the control group, respectively (Figure 6B). Collectively, these findings demonstrate that rtAmR possesses potent osteogenic activity and effectively promotes mineralization and repair of alveolar bone defects.

### 3.6. Immunohistochemical Profiling of the Immune Microenvironment

**Figure 7:**
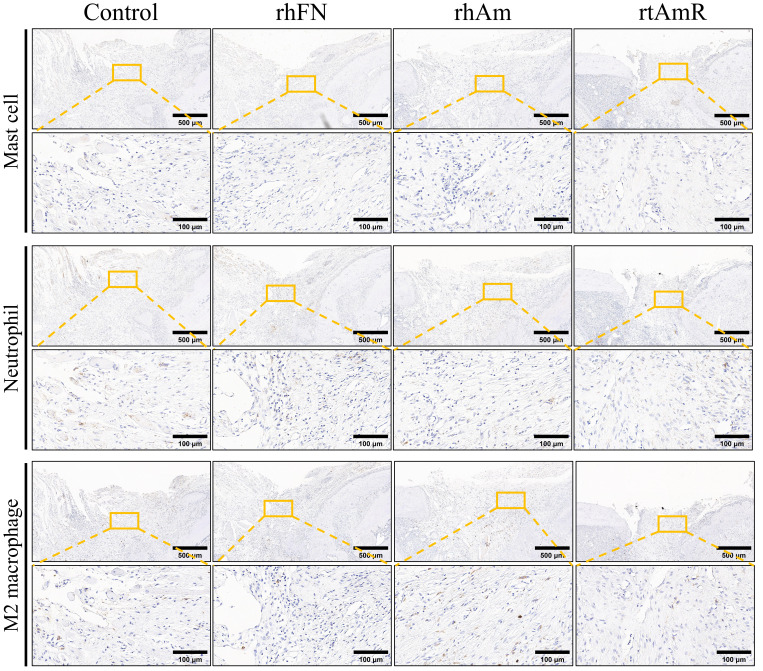
Immunohistochemical analysis of immune cell infiltration in the alveolar bone defect area. Representative images of CD117^+^ mast cells, MPO^+^ neutrophils, and CD206^+^ M2 macrophages in the defect area. Regions marked by yellow rectangles are displayed at higher magnification in the panels to the right.

Immunity is recognized as a key factor in maintaining tissue homeostasis; therefore, evaluating the host immune response to implanted tissue-engineered constructs is of critical importance [35]. As shown in Figure 7, immunohistochemical staining revealed that MPO+ neutrophils and CD117+ mast cells were rarely detected in all groups, with no obvious differences between the rtAmR group and the control group, indicating that rtAmR does not elicit acute inflammation or allergic reactions and exhibits favorable biosafety. Similarly, CD206+ M2 macrophages were also infrequent, and no obvious differences were observed among the groups, suggesting that rtAmR does not promote bone regeneration through an alternative macrophage polarization pathway.

## 4. Discussion

In this study, we designed and constructed a novel recombinant fusion protein, rtAmR, by fusing the highly conserved N- and C-terminal domains of Am with the RGD domain of FN. This biomimetic molecular design aims to yield a dual-functional protein that combines early cell adhesion promotion with osteogenic differentiation induction. In vitro and in vivo experimental results demonstrate that rtAmR not only retains the intrinsic osteoinductive properties of Am but also acquires markedly enhanced cell adhesion capacity, ultimately achieving superior alveolar bone defect repair.

The core finding of this study is that upon replacing the central hydrophobic region of Am with the RGD domain of FN, the fusion protein rtAmR exhibits no antagonism between the two critical functions of cell adhesion and osteogenic differentiation; instead, its pro-osteogenic effect surpasses that of rhAm. We hypothesize that this phenomenon may involve two previously underappreciated mechanisms. On the one hand, the central hydrophobic region is neither the sole determinant nor an absolutely essential domain for the osteogenic activity of Am—its absence is tolerable, and even beneficial, at least in the context of functional compensation provided by the introduced RGD domain. This inference aligns with findings on the natural splice variant LRAP, which likewise lacks the central hydrophobic region yet effectively induces osteogenic differentiation of mesenchymal stem cells [36]. On the other hand, the incorporation of the RGD domain may indirectly amplify the pro-osteogenic signaling of the Am terminal domains by enhancing cell spreading and integrin-mediated signal transduction [37,38].

Furthermore, this study also evaluated the effects of rtAmR on key cells of the immune system. The results showed no significant differences in the numbers of neutrophils or mast cells following rtAmR administration compared with the control group; neutrophils are a critical line of defense in the innate immune system against acute inflammation [39], while mast cells are primarily involved in allergic reactions [40]. These findings indicate that rtAmR does not elicit acute inflammation or allergic responses, demonstrating favorable biosafety. M2 macrophages can secrete IL-10, TGF-β, BMP-2, VEGF and other factors, thereby exerting anti-inflammatory effects and promoting osteogenesis and angiogenesis [41]; however, immunohistochemical staining in the present study revealed that CD206^+^ M2 macrophages were rarely detected in the defect area, with no obvious difference between the rtAmR group and the control group. This suggests that the enhanced bone regeneration induced by rtAmR is unlikely to be mediated through an M2 macrophage-dependent immunomodulatory pathway. Instead, the osteogenic effect of rtAmR is more plausibly attributed to its direct action on osteoprogenitor cells—specifically, the conserved terminal domains of amelogenin provide osteoinductive signals, while the RGD domain promotes cell adhesion and spreading, collectively facilitating bone repair without relying on M2-related immunomodulation.

Compared with bone morphogenetic protein 2 (BMP-2), which is widely used in clinical practice, the recombinant fusion protein rtAmR exhibits notable potential advantages in terms of safety and functional integration. Although BMP-2 possesses potent osteoinductive activity, its clinical application often requires supraphysiological doses that can readily provoke adverse effects such as ectopic ossification, local inflammatory reactions, and even potential tumorigenesis [42]. In contrast, rtAmR comprises Am terminal sequences and an FN-derived RGD domain, a molecular composition that more closely mimics the extracellular microenvironment of dental-derived stem cells. This characteristic theoretically confers superior biocompatibility to rtAmR, potentially circumventing the severe adverse effects associated with BMP-2 [43]. Moreover, the RGD domain carried by rtAmR efficiently mediates cell adhesion, a process critical for the migration and colonization of osteoblasts at defect sites during early bone repair [44]. Experiments confirm that the cell adhesion capacity of rtAmR is comparable to that of rhFN. Thus, this single molecular entity integrates both cell adhesion and osteoinductive functions, enabling efficient cell recruitment without the need for additional exogenous protein carriers.

This study has several limitations that warrant further investigation. First, local injections were employed for animal experiments; although effective in this system, they do not directly support clinical translation. Future efforts should prioritize the development of delivery systems based on sustained release microspheres, thermosensitive hydrogels, or covalent coupling to bone scaffolds to achieve controlled release and prolonged local retention of rtAmR. Second, although we performed immunohistochemical staining to assess immune cells in the defect area at 4 weeks post treatment, the dynamic changes of the immune response throughout the entire treatment course remain to be further investigated. Third, the underlying molecular mechanisms require further dissection, for example, whether conformational cooperativity exists between the RGD domain and the Am domains. Fourth, the in vivo model used in this study was a healthy rat alveolar bone defect model; although it is a standardized system for evaluating osteogenic capacity, it cannot fully recapitulate the chronic, plaque-driven inflammatory pathological microenvironment of human periodontitis. Finally, only hSCAPs were employed as the in vitro cell model; subsequent studies should validate the biological functions of rtAmR in human periodontal ligament stem cells, primary osteoblasts, and multicellular co-culture systems.

In summary, through rational molecular engineering, this study successfully constructed the recombinant fusion protein rtAmR, which integrates the efficient cell adhesion function mediated by the RGD domain of FN with the osteoinductive activity conferred by the conserved terminal domains of Am. This dual-functional molecule significantly promotes the adhesion, spreading, and osteogenic differentiation of hSCAPs in vitro and achieves enhanced alveolar bone defect repair in vivo. This work not only provides a novel candidate protein molecule for the clinical treatment of alveolar bone defects but also offers a valuable strategy for the rational design and construction of multifunctional protein materials in tissue engineering.

## Figures and Tables

**Table 2:** The primer sequences of the target gene for qPCR.

Gene Name	Direction	Primer Sequences (5′–3′)
GAPDH	Forward	ATCAAGAAGGTGGTGAAGCAG
	Reverse	GTCATACCAGGAAATGAGC
RUNX2	Forward	GCGTCAACACCATCATTCTG
	Reverse	CAGACCAGCAGCACTCCATC
OSX	Forward	TCTGCGGGACTCAACAACTC
	Reverse	GCTTGTAAAGGGGGCTGGAT

## Data Availability

The original contributions presented in the study are included in the article, further inquiries can be directed to the corresponding authors.
